# Immigration, Law, and (In)Justice: Coronavirus and Its Impact on Immigration

**DOI:** 10.1177/1057567720951848

**Published:** 2020-12

**Authors:** Krystlelynn Caraballo

**Affiliations:** 1 Andrew Young School of Policy Studies, 1373 Georgia State University, Atlanta, GA, USA

**Keywords:** immigration law, policy, coronavirus-19, supreme court, DACA

## Abstract

This introduction to the *International Criminal Justice Review* provides a brief overview of the books reviewed in this special issue, which address multiple facets of the immigration system. Then, I provide an overview of the key immigration policy changes and legal challenges that have occurred in the midst of the coronavirus (COVID-19) pandemic between March and July 2020. This health crisis exacerbated the struggles faced by the immigrant community and exemplify the systemic barriers that impede integration.

## Introduction

The most impactful thing you can do is bear witness.

—Marty Rosenbluth

For this issue of the *International Criminal Justice Review*, we have curated reviews of books on immigration, law, and (in)justice. This comes at a critical time. Initially slated for June 2020, this issue was delayed as a result of the coronavirus (COVID-19) pandemic. This health crisis exacerbated the systemic struggles faced by the immigrant community. My introduction provides a brief overview of the books reviewed, which address multiple facets of the immigration system. Then, I provide an overview of the key immigration policy changes and legal challenges that have occurred in the midst of the COVID-19 crisis between March and July 2020.

As I detail elsewhere (Caraballo, 2020), immigrants, historically, have been deemed a threat to American jobs, safety, and political advantage. Although repeatedly refuted (Wang, 2012), these perceived threats have shaped immigration policy—often in favor of increased enforcement and restrictive laws (Buckler, 2008). Immigrants are often scapegoated in the wake of large-scale crises including terrorist attacks (Sampaio, 2015), economic downturns (Burns & Gimpel, 2000), and, now, the COVID-19 pandemic (Bieber, 2020).

The books reviewed in this special issue illustrate the complexity of the immigration system and the need for an interdisciplinary understanding of the sociological, political, and legal factors intersecting immigration and social science. This understanding is essential for advocacy and future research. The reviews are grouped by themes in immigration, starting with the impact of political climate, proceeded by enforcement, detention, deportation, resistance, and a foreign case study. These select books provide a holistic overview of the key areas in immigration research.

First, an award-winning book, *Undocumented Fears: Immigration and the Politics of Divide and Conquer in Hazelton, Philadelphia* (Longazel, 2016), highlights the impact of the political climate on the tension between Whites and Latinx communities. The high-profile conflict in this small town illustrates tensions currently occurring across the United States.

The subsequent reviews address police enforcement of immigrant communities and law enforcement agencies’ relationships with the U.S. Immigration and Customs Enforcement (ICE). *Policing Immigrants: Local Law Enforcement on the Front Lines* (Provine et al., 2016) addresses “patchwork policing” in immigration enforcement. This book investigates the perspective and actions of local police departments amid increasing pressure to cooperate with federal agencies, using historical trends, survey research, and case studies.

*Protect, Serve, and Deport: The Rise of Policing as Immigration Enforcement* (Armenta, 2017) focuses on the enforcement practices of the Nashville Police Department. This award-winning ethnography details the interactions law enforcement officials had with the Nashville Latinx immigrant community through observations during ride alongs and interviews with patrol officers and deputized officers in the local jail.

The newest edition of *Law enforcement and the INS: A Participant Observation Study of Control Agents* (Weissinger, 2017) updates prior research that used the perspective of criminal investigators of the Immigration and Nationality Service. This important contribution to longitudinal qualitative research compares those perspectives to the current immigration enforcement priorities implemented post–September 11.

The last enforcement-focused book, *Pathogenic Policing: Immigration Enforcement and Health in the US South* (Kline, 2019), addresses the impact of immigration enforcement policy on immigrants’ physical and mental health and access to health care. The author stresses the negative consequences of restrictive policies, including immigrants’ reliance on a “Shadow Medical System” or fear in seeking medical attention at all. This phenomenon, long established in the medical field (Berk & Schur, 2001; Maldonado et al., 2013), has dire consequences amid the COVID-19 pandemic.

The following books address immigration detention. *Boats, Border, and Bases: Race, the Cold War, and the Rise of Migration Detention in the US* (Loyd & Mountz, 2018) tells the racialized history of the U.S. migration detention system that was developed and expanded to deter Haitian and Cuban migrants during the Cold War, establishing the legal and institutional basis for contemporary detention practices.

*Caging Border and Carceral States: Incarcerations, Immigration Detentions, and Resistance* (Chase, 2019) addresses the interconnection of racial oppression and incarceration in the U.S. South and West, including the detention and deportation of migrants along the U.S.-Mexico border. This essential work situates the historical role of immigration detention on the broader issue of mass incarceration in America.

*Beyond Deportation: The Role of Prosecutorial Discretion in Immigration Cases* (Wadhia, 2015) focuses on how prosecutorial discretion has been used as a form of relief from removal prior to the John Lennon case through President Obama’s Deferred Action for Childhood Arrivals (DACA) Executive Order. Wadhia argues that DACA was an important step in standardizing the granting of prosecutorial discretion for select subgroups of immigrants and improving transparency by establishing criteria by which qualifying individuals could proactively apply. As of March 2020, there were 643,430 active DACA recipients in the United States, with over 825,000 individuals receiving relief since the program’s inception in 2012 (Svajlenka & Wolgin, 2020). The Trump Administration’s attempt to rescind DACA and the resulting Supreme Court decision (discussed below) illustrates the varying degrees to which American presidents have used—or restricted—prosecutorial discretion.

*American Presidents, Deportation and Human Rights Violations: From Carter to Trump* (Hing, 2018) explores the immigration policies and practices over the last 40 years, detailing the circumstances and political response of each Presidential Administration.

*Immigrants Under Threat: Risk and Resistance in Deportation Nation* (Prieto, 2018) shifts the conversation from what has been done to Mexican immigrants to how they respond to and resist repression. This insight shifts the narrative from immigrants as powerless victims of circumstances to active advocates, challenging the social constraints of their agency.

Building on the theme of resistance, award-winning book *Latino Mass Mobilization: Immigration, Racialization, and Activism* (Zepeda-Millán, 2017) focuses on the 2006 protests against the Sensenbrenner bill (H.R. 4437). It is based on 131 interviews with protest organizers and participants combined with other sources of data to examine the influence these demonstrations had on policy.

The last of the resistance books, *Undocumented Storytellers: Narrating the Immigrant Rights Movement* (Bishop, 2018), chronicles the immigration stories of 40 undocumented young adults living in New York City. While some are DACA recipients, others are not, but each one lends their voice to the immigrant rights movement.

*Citizen Outsider: Children of North African Immigrants in France* (Beaman, 2017) demonstrates that the United States is not the only country struggling to adequately address the plight of immigrants. *Citizen Outsider* uses fieldwork and interviews of middle-class children of North African Immigrants in France to highlight the impact of race and ethnicity on marginalization. Each of these remarkable works contributes to broader conversations on immigration, law, and justice matters.

## Immigration Policy and Enforcement Amid COVID-19

In March 2020, as countries across the globe closed their borders to travel, many Americans were skeptical that COVID-19 was any different than the flu or previous health scares. The mood quickly changed as hospitals documented outbreaks. Schools scrambled to transition classes online. Panic buying left grocery stores with bare shelves, and essential items became coveted commodities. On March 14, President Trump declared a national emergency. To combat the spread, most states imposed stay at home orders. Many industries were limited—or closed off—from business as usual. Unemployment skyrocketed.

Since its inception in January 2019, the Migrant Policy Protocols (MPP)—frequently referred to its moniker the “remain in Mexico” policy—has sent thousands of asylum seekers to await their asylum hearings in Mexico or designated U.S. locations. On March 20, 2020, the Department of Homeland Security (DHS) limited all “nonessential” travel across borders, which some referred to as an “asylum ban.” The DHS website defined “nonessential travel” as travel that is considered tourism or recreational but did not include “asylum” or “refugee” as categories under the unaffected travel, leaving asylum seekers in limbo. The Human Rights First (2020) organization wrote a letter urging DHS to end MPP and “parole these individuals into the United States where they can secure shelter and continue their pending proceedings when immigration courts can safely reopen.” Simultaneously, the refugee resettlement program was temporarily suspended by the United Nations High Commissioner for Refugee and the International Organization for Migration from March 19 through June 17, 2020. Despite this temporary suspension, 285 refugees were resettled across the United States (Rush, 2020), and, at the time of this writing, the nonessential travel restriction measures were extended through August 20, 2020.

On April 22, 2020, President Trump issued Proclamation 10014. It suspended entry into the United States by certain foreign nationals who were seeking lawful permanent resident status. On renewing Proclamation 10014 in June 2020, several nonimmigrant (temporary) visas—including H-1B, H-2B, J, and L visas (and their accompanying family members)—were also suspended, citing the high unemployment rate resulting from public health measures to reduce the spread of COVID-19 (Kandel, 2020; Wilson, 2020).

Given the international response, these actions may initially seem reasonable. However, because of President Trump’s stance on immigration and his divisive rhetoric, many criticized his response as merely capitalizing on the pandemic as a justification to bypass established procedures for enacting immigration reform.

Although admissions virtually ceased, detention and removals continued. As states contemplated ways to reduce the spread in close-quarter institutions, including jails and prisons, United Nations agencies on human rights, global health, migrants, and refugees criticized ICE’s (non)response to the pandemic. Amnesty International (2020) argued that the threat of an outbreak merited the release of asylum seekers and those with preexisting conditions. Likewise, the American Civil Liberties Union sued ICE on behalf of detainees at multiple detention centers across the country, seeking detainees’ immediate release due to underlying medical conditions or age. These factors placed them at high risk of serious illness or death in the event of COVID-19 infection. The lawsuits argued that transmission was “exceedingly likely,” given facilities crowded and unsanitary conditions and inability to maintain social distancing. Additionally, removals continued, and several countries have attributed increases in their COVID-19 cases to deportees who were not consistently tested for symptoms in the United States (Sullivan et al., 2020).

## Supreme Court Rulings and Pending Litigation

### DHS v. Regents of the University of California

On June 15, 2012, the DHS issued the “Exercising Prosecutorial Discretion With Respect to Individuals Who Came to the United States as Children” memo, hereafter referred to as the DACA program, after Congress failed to pass the Development, Relief and Education for Alien Minors Act. On September 5, 2017, the Trump Administration announced the decision to phase out the DACA program.

During oral arguments, the government relied on the precedence set by *United States v. Texas* (2016) to assert that the decision to phase out the DACA program did not violate the Administrative Procedural Act (APA). In that case, the Court of Appeals for the U.S. Fifth Circuit affirmed that the Deferred Action for Parental Arrivals (DAPA) executive order violated the APA, and the program violated the Take Care Clause. Concerning DACA, the government reasoned that the decision is not subject to judicial review, based on the “Chaney Presumption.” This presumption, based on *Heckler v. Chaney* (1985), argues that decisions by administrative agencies not to exercise enforcement actions are precluded from judicial review. They argued that judgment, in effect, ended a previous nonenforcement policy whereby the DHS agreed not to enforce the Immigration and Nationality Act of 1952 against hundreds of thousands of illegal aliens. Additionally, they argued, DACA was a temporary stopgap measure that, on its face, could be rescinded at any time.

The respondents argued that the termination of DACA was subject to judicial review. Also citing the Chaney case, they wrote, “When an agency does act to enforce, the action itself provides a focus for judicial review because it imposes the coercive power of the government with respect to individual liberty and property.” (p. 54). They further argued government termination of DACA triggered “abrupt, tangible, adverse consequences and substantial disruptions in the lives of 700,00+ individuals, their families, employers, communities, and Armed Forces.” The respondents asserted that these “reliance interests” were not adequately considered, and the decision was unduly influenced by the Attorney General’s statement that the implementation of DACA was “illegal.” They further argued that DACA differed from DAPA in that there was no guaranteed path to residency or citizenship.

On June 18, 2020, the Supreme Court held in a 5-4 decision that the DHS’s reasoning for rescinding DACA was inadequate and, therefore, violated the APA. This decision allows current DACA recipients, and eligible childhood arrivals, to receive continued relief from removal and work authorization. Many people celebrated this decision as a victory. However, this ruling does not prevent the Trump Administration—or future administrations—from taking new action to rescind DACA. The Supreme Court’s opinion reaffirms that a presidential administration has the power to end DACA but must supply adequate justification for doing so under the APA. Specifically, the Supreme Court reasoned that DHS should have at least explored the possibility of granting DACA recipients protection from removal, even if terminating their eligibility for benefits; the DHS failed to consider the myriad ways in which DACA recipients and those connected to them have come to rely on the program for their educational, professional, and personal endeavors. Although the APA does not require DHS to accommodate these “reliant interests,” they must be considered before rescinding the program.

The Supreme Court decision indeed came at a critical time. Amid the pandemic, more than 200,000 DACA recipients were classified as “essential workers,” 29,000 of whom worked in the health care field (Prchal Svajlenka & Wolgin, 2020). Although the pandemic had no influence on the arguments presented before the Supreme Court, proponents of DACA used this information to garner public support for immigration reform that would provide pathways to citizenship for individuals who met select criteria and have contributed to the United States.

### DHS et al. v. Vijayakuma Thuraissigiam

Soon after the DACA decision, the Supreme Court ruled against an asylum seeker who sought a second opportunity to request asylum. According to the Supreme Court documents, Respondent Vijayakumar Thuraissigiam, a Sri Lankan national, was stopped 25 yards from the southern border at an unauthorized point of entry and without an entry document. He was detained for expedited removal. According to court documents, he claimed a fear of returning to Sri Lanka

because a group of men had once abducted and severely beaten him, but he said that he did not know who the men were, why they had assaulted him, or whether Sri Lankan authorities would protect him in the future. He also affirmed that he did not fear persecution based on his race, political opinions, or other protected characteristics. (p. 9)

Based on this information, an asylum officer rejected his credible fear claim, then a supervising officer agreed, an immigration judge affirmed. Thuraissigiam then filed a federal habeas petition, asserting a fear of persecution based on his Tamil ethnicity and political views, requesting a new opportunity to apply for asylum. The District Court dismissed the petition, but the Ninth Circuit reversed that decision, holding that, as applied in this case, §1252(e)(2) violates the Suspension Clause and the Due Process Clause.

The Supreme Court rejected Thuraissigiam’s claims. In short, they ruled that §1252(e)(2) does not violate the Due Process Clause. More than a century of precedent establishes that, for foreigners seeking initial entry, “the decisions of executive or administrative officers, acting within powers expressly conferred by Congress, are due process of law.” (p. 3). They also rejected Thuraissigiam’s argument that this rule does not apply to him because he succeeded in making it 25 yards into U.S. territory. The Court reasoned:

[T]he rule would be meaningless if it became inoperative as soon as an arriving alien set foot on U.S. soil. An alien who is detained shortly after unlawful entry cannot be said to have “effected an entry.” *Zadvydas v. Davis*, 533 U.S. 678, 693. An alien in respondent’s position, therefore, has only those rights regarding admission that Congress has provided by statute. In respondent’s case, Congress provided the right to a “determin[ation]” whether he had “a significant possibility” of “establish[ing] eligibility for asylum,” and he was given that right. (p. 35)

This case reaffirms asylum officers’ power in granting and denying “credible fear” determinations. Also, the court reaffirmed the federal court’s limited ability to review a petition for a writ of habeas corpus, as outlined by the Illegal Immigration and Immigrant Responsibility Act (IIRIRA) of 1996. Specifically, IIRIRA states that courts may not review “the determination” that an applicant lacks a credible fear of persecution. This demonstrates the need for asylum applications to be fully detailed and articulated prior to the initial asylum interview. Asylum seekers who are represented have somewhat greater success in defending their claims. However, the United States has increasingly denied asylum applications since President Trump’s election in November 2016 (Transnational Records Clearinghouse [TRAC], 2020b). Unknown is whether the outcome of his initial application would have been changed by including the claim that the respondent feared persecution based on his ethnicity and political views. The Court highlights that aliens subject to expedited removal have opportunities at three levels to obtain an asylum hearing unless the applicate does not assert a credible fear claim.

### *Plaintiffs v. Steven T. Mnuchin and United States of America*: Civil Action No. 8:20-cv-1148

Scholars have addressed the “collateral consequences” of immigration enforcement on immigrant families and communities including U.S.-born children, spouses, and neighborhoods. During the pandemic, there have been several examples of this spillover effect. One such example involves the stimulus checks sent to Americans amid mass unemployment. The language of the law resulted in U.S. citizen children who have only noncitizen parents or one noncitizen parent and one citizen parent being excluded from receiving the US$500 economic impact payments intended for their benefit. Specifically, the Migration Policy Institute reported that 3.7 million children and 1.7 spouses—who were either U.S. citizens or green card holders—were excluded from the Coronavirus Aid, Relief, and Economic Security (CARES) Act aid unless the parents filed a 2019 tax return as “married filing separately” rather than “married filing jointly,” which can increase the family’s tax burden. This resulted in immigrant families and communities being denied financial support during a global crisis (Chishti & Bolter, 2020). In May 2020, a class-action lawsuit was filed with a Maryland court claiming discrimination in violation of the equal protection principle embodied in the Fifth Amendments’ Due Process Clause. Plaintiffs allege that this scheme intentionally discriminates against U.S. citizen children solely because of their parents’ alienage and discriminates against U.S. citizens who chose to marry a noncitizen.

The government’s response, construed as a motion to dismiss, argued the plaintiffs lacked standing, their case failed to state a claim, and the Maryland court lacked jurisdiction. On June 19, 2020, the presiding judge, Paul W. Grimm, disagreed with the government’s letter and denied their motion to dismiss. He stated, “I find that Plaintiffs have established Article III and statutory standing, that this Court has subject matter jurisdiction over their claims, and that they have adequately alleged an equal protection claim.” The judge’s ruling allows the lawsuit to proceed. At the time of this writing, the outcome of the case remains to be determined as well as whether individuals will be excluded from future stimulus checks.

## Conclusion

This introduction began by quoting immigration lawyer and activist, Marty Rosenbluth. To “bear witness” means to demonstrate that something exists or is true. The books reviewed in this issue bear witness to the causes, nature, and consequences of immigration law and (in)justice. None of those books could have reasonably predicted a global pandemic; however, the underlying circumstances predicating the COVID-19 response to immigration were predictable based on historical immigration policy, immigrants as perceived threats to public safety and economic standing, and the political climate fostered by the recent Presidential Administrations. The books reviewed herein describe this precedent.

Rosenbluth’s passionate keynote address calling for us to “bear witness” extends beyond merely understanding the phenomenon from an academic lens but rather emphasizes interaction with those most impacted by immigration policy as well as those involved in the enactment and enforcement. Bearing witness involves conversations with those most affected, who are often left out of dialogue that impacts them directly, engaging with law enforcement regarding their interactions with immigrant communities, observing immigration courts and taking note of procedural injustices, and ensuring that we hold agencies accountable for their policies, procedures, and transparency with the public. Many scholars, activists, and organizations have long led the charge on these initiatives, but systemic change requires immense backing and tenacity.

Take, for example, findings of the TRAC, a nonpartisan agency that collects immigration data through the use of Freedom of Information Act requests. In TRAC’s October 2019 and November 2019 reports, they found 1,507 and 3,799 missing applications for relief from removal, respectively. TRAC (2020a) repeatedly wrote to EOIR Director James McHenry requesting information on why these records were missing from their files and to provide documentation for missing applications. They state, “[n]ot only have these cases disappeared entirely, they have not been restored in any subsequent data releases and the number of missing relief applications continue to grow.” Indeed, they report:

the number of relief applications that were present in the March 2020 data release but were missing in the April release jumped to 68,282. This is just the number of records that disappeared over a single month. It does not include the ever growing number of applications that had previously disappeared month-by-month. As was true in past months, roughly four out of five of the records in the March 2020 release that disappeared from April’s release concerned applications on which the court had rendered its decision, including many cases in which the immigration judge had granted asylum as well as other forms of relief.

To put that into perspective, the number of missing cases just last month is more than the 63,734 asylum applications received by the Immigration Courts during all of FY 2015….

In a time when most people are distracted by the ongoing pandemic and the repercussions it has had on their lives, it is vital not to lose sight of the structural injustices that continue, particularly in vulnerable communities. TRAC (2020a) reports that after multiple attempts, they received a reply from the EOIR Executive Director, but he denied any data discrepancies and “attacked” the agency’s motivations for requesting additional data.

Some people argue that the COVID-19 pandemic is an extraordinary circumstance, so not reflective of immigration affairs more generally. Others, as I have done herein, argue that the pandemic is another example of how governments use crises to accelerate nativist policies and preexisting, anti-immigrant agendas. The COVID-19 pandemic has illustrated the various ways in which citizenship is prioritized over human rights. Some policies would have actively put foreign nationals in harm’s way, including a proposed rule in which international students would have their status terminated if they did not attend in-person classes. Pre-COVID-19 guidelines required international students to take no more than a single online class, but given the mass transition to distant learning, the policy was condemned by U.S. Universities. Harvard University and MIT filed lawsuits to halt the implementation of the policy and DHS soon rescinded the requirement.

As scholars, it is imperative that we are not only aware of the historical implications of immigration policies but also that we are also familiar with the local, state, and federal policies that govern. Impactful research must account for the interwoven intricacies that elicit a chain reaction of events. In order to achieve systemic change, we too must bear witness and continue to engage in interdisciplinary, collaborative work.
